# Enzymatic Activity Enhancement of Non-Covalent Modified Superoxide Dismutase and Molecular Docking Analysis

**DOI:** 10.3390/molecules17043945

**Published:** 2012-03-30

**Authors:** Yan-Zi Qiu, Zong-Hua Huang, Fa-Jun Song

**Affiliations:** College of Life Science/Key Laboratory for Biotechnology of the State Ethnic Affairs Commission, South Central University for Nationalities, 708 Minyuan Road, Wuhan 430074, China; Email: qiuyan862724@163.com (Y.-Z.Q.); hzhlove55@yahoo.cn (Z.-H.H.)

**Keywords:** non-covalent modification, superoxide dismutase, hydroxypropyl-β-cyclo-dextrin, fluorescence intensity, molecular docking

## Abstract

The enzyme activity of superoxide dismutase was improved in the pyrogallol autoxidation system by about 27%, after interaction between hydroxypropyl-β-cyclo- dextrin and superoxide dismutase. Fluorescence spectrometry was used to study the interaction between hydroxypropyl-β-cyclodextrin and superoxide dismutase at different temperatures. By doing this, it can be found that these interactions increase fluorescence sensitivity. In the meantime, the synchronous fluorescence intensity revealed the interaction sites to be close to the tryptophan (Trp) and tyrosine (Tyr) residues of superoxide dismutase. Furthermore, molecular docking was applied to explore the binding mode between the ligands and the receptor. This suggested that HP-β-CD interacted with the B ring, G ring and the O ring and revealed that the lysine (Lys) residues enter the nanocavity. It was concluded that the HP-β-CD caused specific conformational changes in SOD by non-covalent modification.

## 1. Introduction

**Figure 1:**
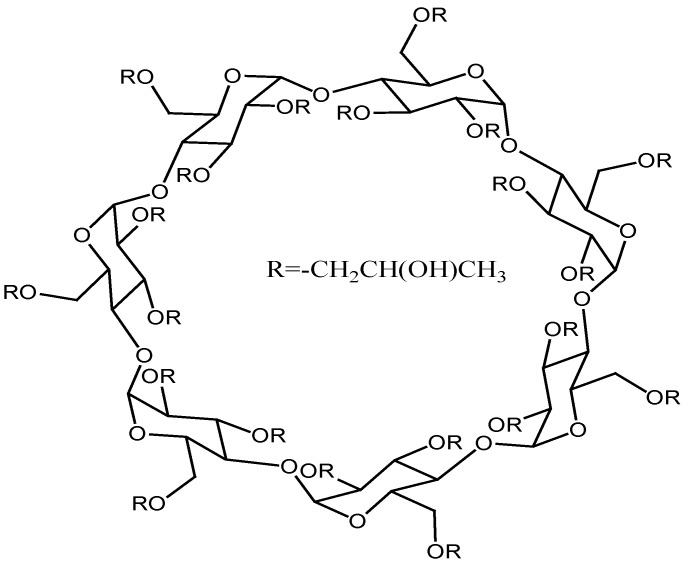
The structure of HP-β-CD.

Cyclodextrins (CDs) are truncated-cone polysaccharides mainly composed of six to eight D-glucose monomers linked by α-1,4-glucosidic bonds. They have a hydrophobic central cavity and hydrophilic outer surface and can encapsulate model substrates to form host-guest complexes or supermolecular species [1]. It is well-known that cyclodextrins form noncovalent inclusion complexes with a wide variety of lipophilic drug molecules by taking up a whole molecule, or some part of it into the cavity, with the stability of the resulting complex depending on different contributions, such as van der Waals interactions, hydrophobic effects, solvent reorganization, hydrogen bonding, key-lock principle, *etc*. [2,3]. Hydroxypropyl-β-cyclodextrin (HP-β-CD; Figure 1) has been extensively investigated for its relatively high water solubility, low toxicity, and satisfactory inclusion ability [4,5,6,7,8,9,10].

Human beings can’t live without oxygen. The cells which rely on oxygen as the final acceptor of electrons in respiration, allow them to extract far more energy from food than that would be possible without oxygen. However oxygen is also a kind of dangerous chemical substance. The superoxide anion radical (O_2_^•−^) is an inevitable and cytotoxic byproduct of aerobic metabolism [11,12,13]. To combat the adventitious production of this ROS, all aerobes possess metalloenzyme defense systems known as superoxide dismutases (SODs) that catalyze the disproportionation of O_2_^•−^ to H_2_O_2_ and O_2_ through alternate oxidation and reduction of their respective metal centers [14,15,16]. With the development of science and technology, some new methods have been used to research the structure of SOD, such as X-ray diffraction and solution NMR [17,18,19]. As an additive, hydroxy-propyl-β-cyclodextrin can interact with proteins or other biological macromolecules. Spectroscopy has been used to research the protein-ligand interactions with notable results [20,21,22]. This work aimed to investigate the interactions between hydroxypropyl-β-cyclodextrin and superoxide dismutase. We use the optimized methods of pyrogallol autoxidation [23] and fluorescence spectrometry [24,25] to inspect the rate of oxidation reaction rate inhibition and non-covalent modifications. In order to investigate the sites of protein-ligand interaction, HP-β-CD was selected as a reference and docked into the binding site of SOD. 

## 2. Results and Discussion

### 2.1. Enzymatic Activity Results

**Figure 2:**
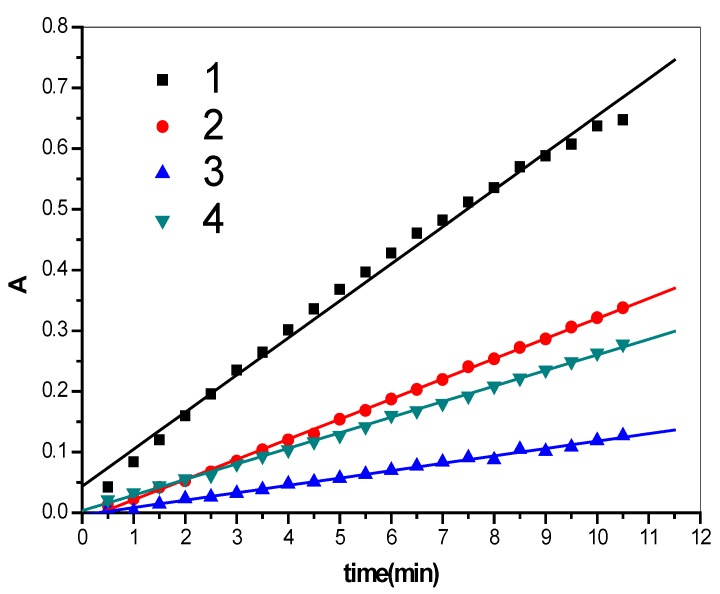
The results of the oxidation under the different conditions (line 1: Oxidation rate of pyrogallol; line 2: The concentration of SOD is 1 × 10^5^ U/L; line 3: The concentration of SOD is 2 × 10^5^ U/L; line 4: The SOD is modified by HP-β-CD).

According to the enzyme activity results, it can be found that when the concentration of SOD is too high, the pyrogallol oxidation reaction doesn’t happen; Lines 2 and 3 in Figure 2 prove that when the concentration of SOD is 1 × 10^5^ U/L and 2 × 10^5^ U/L, pyrogallol oxidation reactions occurred, and the inhibition effect of SOD is also obvious. The light absorption value becomes a stable linear relationship within about 10 min. Experiments show that different concentrations of HP-β-CD with SOD give different values for the oxidation reaction inhibition rate. When the response time was more than 4 h the values were unchanged. In line 4, it can be demonstrated that the changed absorption value is 0.0257 per minute.

To sum up, when SOD is present, the oxidation reaction of pyrogallol is restrained. From Table 1 it can be found that when SOD and HP-β-CD form an inclusion complex completely, the inhibition is enhanced, and the inhibitory rate is about 58%, which is the 1.27 times as high as with SOD alone. That it, when SOD is encapsulated, the enzyme activity of SOD improved 1.27 times.

**Table 1:** The regression equation of the oxidation under the different conditions.

Number	Regression equation	r	SD
Line 1	y = 0.0437 + 0.0611x	0.9959	0.0171
Line 2	y = −0.0117 + 0.0332x	0.9996	0.0032
Line 3	y = −0.0036 + 0.0122x	0.9968	0.0031
Line 4	y = 0.0035 + 0.0257x	0.9993	0.0032

### 2.2. Fluorescence Spectrum Experimental Results

#### 2.2.1. Fluorescence Emission Spectra

**Figure 3:**
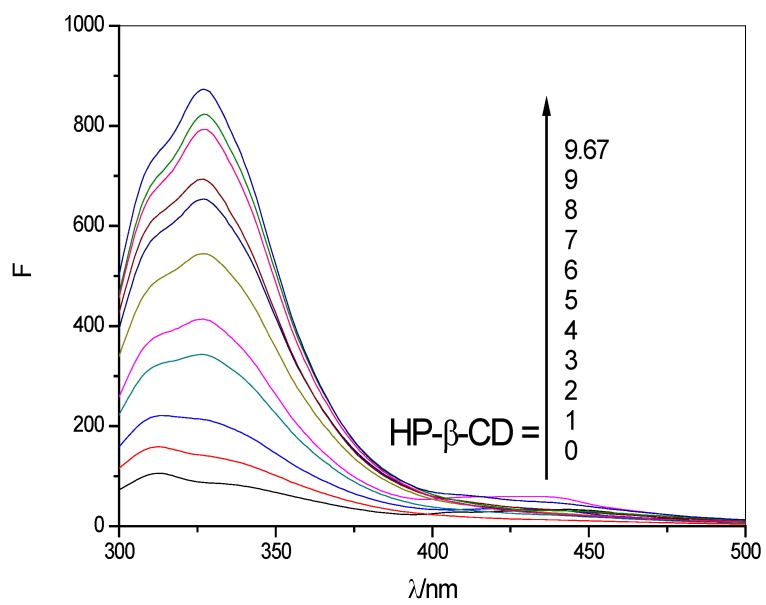
Fluorescence emission spectra of SOD (1 × 10^4^ U/L) at various concentrations of HP-β-CD (λ_ex_ = 280 nm); the concentrations of HP-β-CD: (0–9, 9.67) × 10^−3^ mol/L).

**Figure 4:**
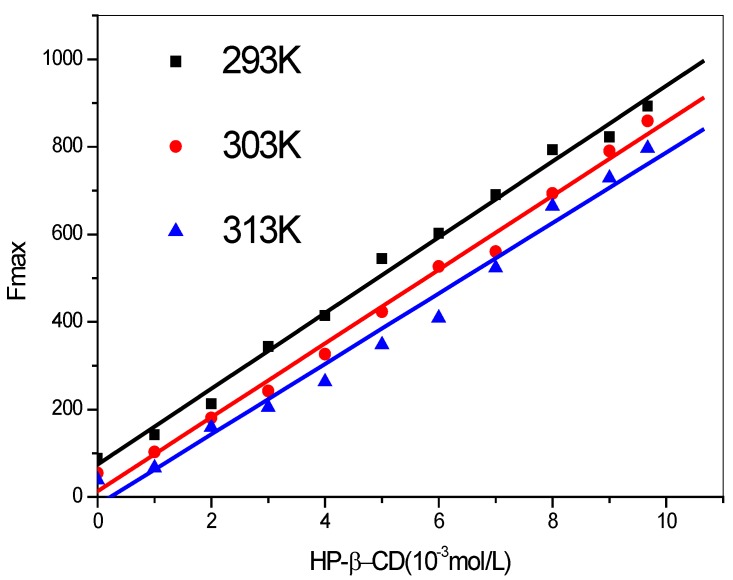
Enhancement in fluorescence intensities (F_max_) of SOD (1 × 10^4^ U/L) with increasing concentration of of HP-β-CD at different temperatures (λ_ex_ = 280 nm).

Figure 3 present the fluorescence emission profiles of SOD at various concentrations of HP-β-CD. Addition of HP-β-CD’s results in significant increases in the fluorescence intensity and the linear regression equations at different temperatures are shown in Figure 4 and Table 2. Such enhanced emission yields are indicative of the penetration of SOD into the relatively less polar cavities of HP-β-CD resulting in the formation of SOD:HP-β-CD inclusion complexes. 

**Table 2:** The regression equation of F_max_ is bound up with the different concentration of HP-β-CD at different temperatures.

T/K	Regression equation	r	SD
293	y = 73.9753 + 86.5676x	0.9967	24.3745
303	y = 13.2668 + 84.3740x	0.9959	26.3180
313	y = −17.9835 + 80.5136x	0.9902	39.1664

For a 1:1 complex formation between fluorescent guest molecules and HP-β-CD the binding constants can be obtained from the data of fluorescent intensity for substituting values of SOD in the Benesi–Hildebrand equation [26]:


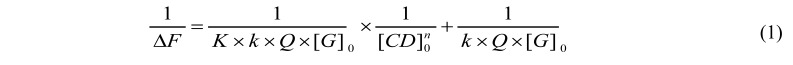


where ΔF = F − F_0_, F and F_0_ represent the fluorescence intensities of SOD in the presence and absence of total added cyclodextrin concentrations, respectively, and n represents the stoichiometry of the complex formed (n = 1 for a 1:1 complex). K is the binding constant for the 1:1 complex. [G]_0_ is the initial concentration of SOD and [CD] the concentration of HP-β-CD, k is an instrumental constant and Q is the fluorescence quantum efficiency.

**Figure 5:**
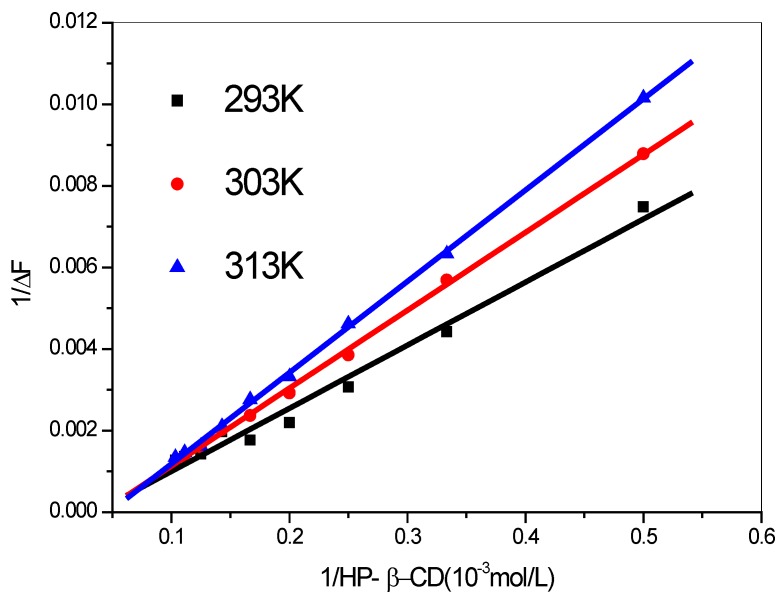
Double-reciprocal plots using steady state fluorescence intensity data for encapsulation of SOD in HP-β-CD at different temperatures.

The plot of 1/ΔF *vs*. 1/HP-β-CD displayed in Figure 5 and Table 3 shows good linearity. This indicates formation of inclusion complexes between the hosts HP-β-CD and the guest (SOD) with a stoichiometry of 1:1 (SOD:HP-β-CD). The binding constants K were found to be 354.5 M^−1^, 408.7 M^−1^ and 473.6 M^−1^ at 293 K, 303 K and 313 K respectively. Additionally, The Gibbs free energy change (ΔG) values are estimated as 14.29 kJ/mol, 15.14 kJ/mol and 16.02 kJ/mol, respectively, for SOD: HP-β-CD complexes at different temperatures.

**Table 3:** The regression equation of double-reciprocal plots and the constant K and thermodynamic parameters ΔG at different temperatures.

T/K	Regression equation	r	K(M^−1^)	ΔG/(kJ/mol)
293	y = −5.4840 + 0.0155x	0.9914	354.5	−14.29
303	y = −7.8066 + 0.0191x	0.9994	408.7	−15.14
313	y = −10.60 + 0.0224x	0.9996	473.6	−16.02

#### 2.2.2. Synchronous Fluorescence Spectra

By setting the excitation wavelength and emission wavelength in a certain wavelength spacing, and using a synchronous scanning excitation and emission monochromator, one may obtain the synchronous fluorescence intensities. Synchronous fluorescence spectra of proteins have already been used to estimate protein conformational changes [27]. The synchronous fluorescence spectral characteristics showed tyrosine residues and tryptophan residues, respectively, when Δλ = 20 nm and Δλ = 80 nm respectively. 

**Figure 6:**
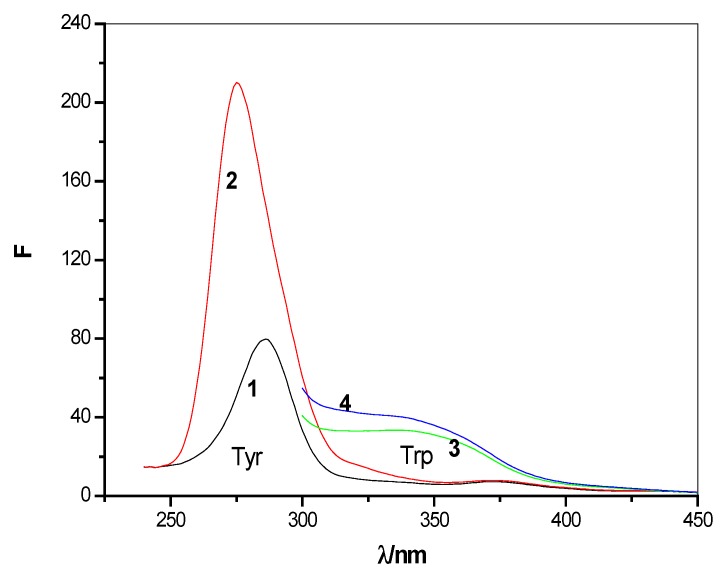
Synchronous fluorescence intensity of SOD and encapsulation of SOD in HP-β-CD; 1,3-SOD (1 × 10^4^ U/L); 2,4-encapsulation SOD in HP-β-CD (1 × 10^−3^ mol/L); 1,2 − Δλ = 20 nm; 3,4 − Δλ= 80 nm.

Like other proteins, superoxide dismutase (SOD)’s tyrosine (Tyr) and tryptophan (Trp) residues, have specific fluorescence absorption peaks. Synchronous fluorescence spectrometry is used to study the interactions between HP-β-CD and SOD. In Figure 6, curve 1 and curve 2 show the synchronous fluorescence spectra of tyrosine (Tyr) residues in the presence and absence of added HP-β-CD, respectively. Curve 2 and curve 4 show the synchronous fluorescence spectra of tryptophan (Trp) residues in the presence and absence of added cyclodextrin concentrations, respectively. The tyrosine (Tyr) and tryptophan (Trp) residues have emission peaks at 280 nm and 340 nm, respectively, and the absorption peaks increased after adding HP-β-CD. In the experiment, from Figure 6, it can be found that the fluorescence intensity of SOD mainly derived from tyrosine (Tyr) residues and the biggest absorption peak position is slight red shifted; conversely, the synchronous fluorescence spectra of tryptophan (Trp) residues shows a blue shift.

### 2.5. Docking Analysis

The Molecular Computer Aided Design Discovery Studio Visualizer 2.5 and MOLCAD programs are used to visualize the binding modes between the ligand and protein. MOLCAD calculates and displays the surfaces of channels, ribbon, and cavities, as well as the separating surface between protein subunits. MOLCAD program provides several ways of creating a molecular surface. Other parameters were established by software defaults.

**Figure 7:**
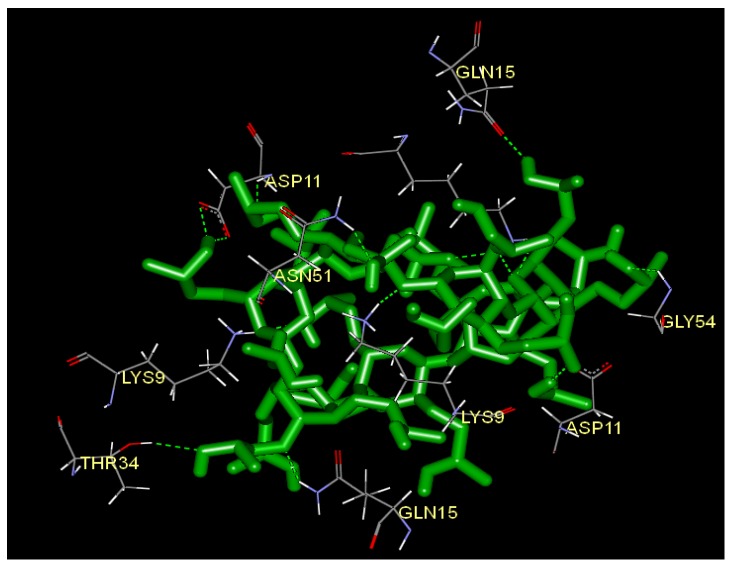
The binding mode between HP-β-CD and the allosteric site of SOD (PDB code ISDA), and key residues and hydrogen bonds were labeled (Discovery Studio Visualizer 2.5).

Molecular docking studies offer precise information for studying the interactions between the ligand and the protein residue. Since the crystal structure of SOD was known, we docked HP-β-CD into the allosteric site of SOD (PDB code ISDA). Figure 7 shows the hydrogen bond donor and hydrogen bond acceptor contour map. Detailed descriptions are given in Table 4. 

**Table 4:** Descriptions of each hydrogen bond along with bonding distance.

Name	Donor Atom	Accepter Atom	Hydrogen bond distance (Å)
:HP:H-B:ASP11:OD1	H	OD1	2.27962
:HP:H-B:ASP11:OD2	H	OD2	2.00114
:HP:H-B:GLN15:OE1	H	OE1	2.02632
:HP:H-G:ASP11:OD1	H	OD1	2.01827
B:ASN51:HD21-:HP:O	HD21	O	1.94949
B:ASP11:H-:HP:O	H	O	2.42934
B:LYS9:HZ1-:HP:O	HZ1	O	1.77084
B:LYS9:HZ1-:HP:O	HZ1	O	2.35214
B:LYS9:HZ2-:HP:O	HZ2	O	2.40027
B:LYS9:HZ2-:HP:O	HZ2	O	1.72237
B:LYS9:HZ3-:HP:O	HZ3	O	1.47502
G:GLN15:HE22-:HP:O	HE22	O	1.8719
G:GLY54:H-:HP:O	H	O	2.16128
G:LYS9:HZ1-:HP:O	HZ1	O	1.34714
O:LYS9:HZ3-:HP:O	HZ3	O	2.07259
O:THR34:HG1-:HP:O	HG1	O	2.34039

**Figure 8:**
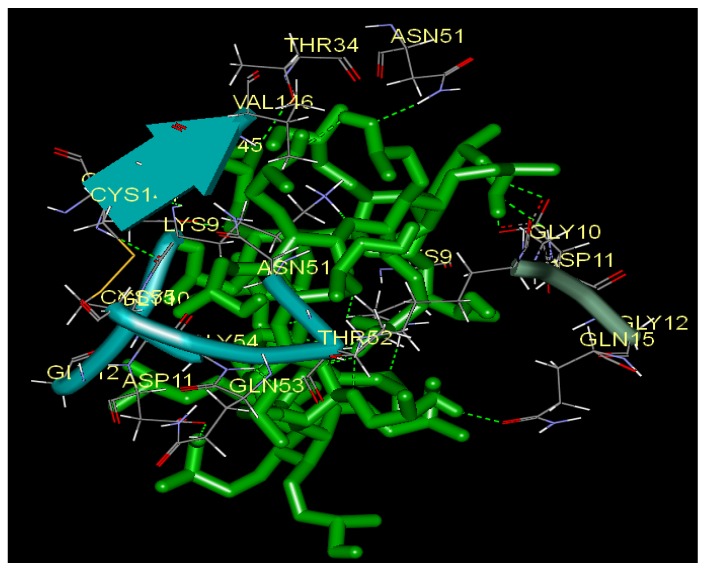
The ribbon surfaces of the allosteric site of SOD (PDB code ISDA) within the HP-β-CD (B ring is light sea green and G ring is sea green. Two disconnected amino acids LYS9 and THR34 can be seen on the O ring. Discovery Studio Visualizer 2.5).

To better visualize the protein structure, the ribbon program for protein residues was used (Figure 8). In this picture, the protein backbone is drawn as a ribbon or tube. Representations of proteins in Richardson style use arrows for beta sheets, cylinders for alpha helices, and tubes for coils and turns. As shown in Figure 8, two rings can be seen (the colour of B ring is light sea green and G ring is sea green). There are two disconnected amino acids on the O ring, which interact with HP-β-CD, so the O ring can’t be seen directly in Figure 8.

**Figure 9:**
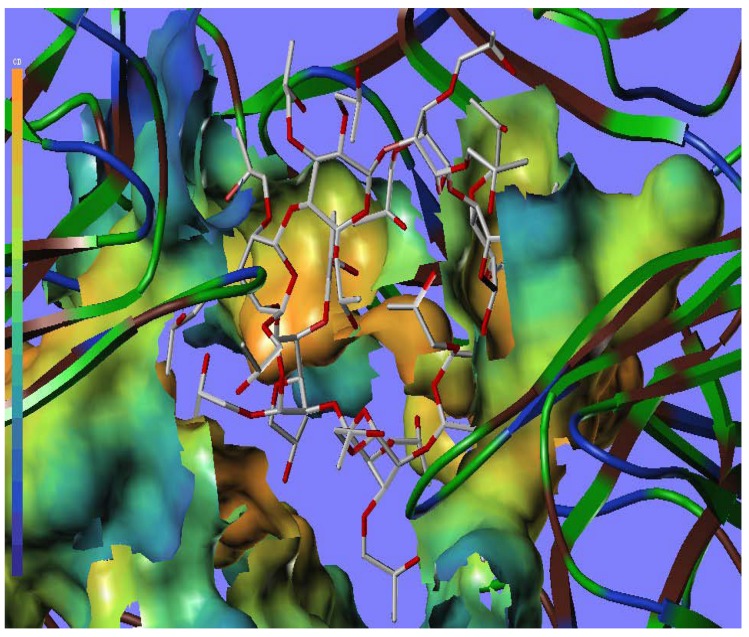
MOLCAD cavity depth potential surface (light red colors denotes the lowest depth) of the allosteric site of SOD (PDB code ISDA) within bound HP-β-CD (color figure online).

Furthermore, to check and corroborate the use of docking, the MOLCAD surface with cavity depth potential was generated and shown in Figure 9. The cavity depth measures how deep a surface point is located inside a cavity of a molecule. The cavity depth color ramp ranges from blue (low depth values represent outside of the molecule) to light red (high depth values represent cavities deep inside the molecule). It can be observed that the binding sites were in the light red region (Figure 9), suggesting that HP-β-CD was placed well in the allosteric site. 

## 3. Experimental

SOD and hydroxypropyl-β-cyclodextrin were purchased from Sigma (Shanghai, China). The SOD’s enzymatic activity is 3000 U/mg. All the solutions are stored at 4 °C and used within a week. Tris-HCl buffer (pH = 8.0) was used in the enzyme activity assay experiments. Tris-HCl (pH = 7.2) buffer was prepared for the fluorescence spectrometric analysis. 

### 3.1. Enzymatic Activity Assay

A stock solution of SOD with a concentration of 3 × 10^5^ U/L and the stock solution of hydroxypropyl-β-cyclodextrin with a concentration of 1 × 10^−2^ mol/L were prepared in phosphoric acid buffer. Acording to Table 5, suitable amounts of solutions were placed in four tubes, and then the changes of absorption value (λ = 325 nm) were recorded in 10 min. 

**Table 5:** Sample components for enzymatic activity assay.

Sample number	1	2	3	4
Tris-HCL/mL	2.991	2.98	2.969	2.98
Pyrogallol/mL	0.009	0.009	0.009	0.009
SOD/mL	--	0.011	0.022	0.022
HP-β-CD/mL	--	--	--	0.1

### 3.2. Fluorescence Spectrometric Analysis

The concentration of SOD was 3 × 10^5^ U/L; the concentration of HP-β-CD was 1 × 10^−2^ mol/L. According to the Table 6, various solutions were mixed and then the fluorescence intensity (λex = 280 nm) was studied. The buffer (0.2 mol/L Tris-HCl) was used as the blank background. Using the synchronous fluorescence spectrum method the tyrosine (Δλ = 20 nm) and tryptophan (Δλ = 80 nm) residues were identified, respectively. 

**Table 6:** Sample components for fluorescence spectra.

Sample number	1	2	3	4	5	6	7	8	9	10	11
SOD/mL	0.1	0.1	0.1	0.1	0.1	0.1	0.1	0.1	0.1	0.1	0.1
HP-β-CD/mL	0	0.3	0.6	0.9	1.2	1.5	1.8	2.1	2.4	2.7	2.9
Tris-HCl/mL	2.9	2.6	2.3	2.0	1.7	1.4	1.1	0.8	0.5	0.2	0

### 3.3. Docking Study

Structures of entire sets of HP-β-CD were generated by the SYBYL 1.10 program package of Tripos. The crystal structure of SOD obtained from Protein Data Bank (PDB ID-ISDA). HP-β-CD was also optimized by a semi-empirical method (MOPAC) as a starting structure in the docking study. 3D structure of the inclusion complex was constructed by using the Sketch molecule module. Other parameters were established by the software defaults. All ligands and water molecules have been removed and the polar hydrogen atoms were added. Protocol, an idealized representation of a ligand that makes every potential interaction with the binding site, was used to guide molecular docking. The automatic docking was applied in this article.

## 4. Conclusions

This research work has compared the different activities of SOD and its inclusion complexes. It can be demonstrated that the complex activities are improved about 27% more than SOD. By testing the inherent fluorescence of SOD, a comparative assessment of the properties of these complexes at different concentrations of HP-β-CD was made. Furthermore, the results of synchronous fluorescence intensity showed the interaction sites of HP-β-CD and SOD close to the tyrosine (Tyr) and tryptophan (Trp) residues. Molecular docking studies and other relevant calculations suggest that HP-β-CD located in the combination region of the two ligands, and interact with the two ligands at the same time. It interacts with the residues, such as Lys9, Asp11, Gln15 and Asn51 of the B ring, Lys9, Gln15 and Gln54 of the G ring and the O ring’s Lys9 and Thr34, and it also revealed that the residues of Lys enter the HP-β-CD nanocavity. Further, HP-β-CD causes specific conformational changes of SOD. Overall, the correlation of the results obtained from these experiments can serve as a useful guideline for the further research in cyclodextrins interact with enzyme protein.
